# Primiparous women’s knowledge of diastasis recti abdominis, concerns about abdominal appearance, treatments, and perceived abdominal muscle strength 6–8 months postpartum. A cross sectional comparison study

**DOI:** 10.1186/s12905-022-02009-0

**Published:** 2022-11-02

**Authors:** Sandra Gluppe, Marie Ellström Engh, Kari Bø

**Affiliations:** 1 Department of Sports Medicine, Norwegian School of Sports Sciences, Sognsveien 220, 0806 Oslo, Norway; 2 Department of Obstetrics and Gynaecology, Akershus University Hospital, Sykehusveien 25, 1478 Lørenskog, Norway; 3 Faculty of Medicine, University of Oslo, Oslo, Norway

**Keywords:** Appearance, Diastasis Recti Abdominis, Function, Health, Postpartum

## Abstract

**Background:**

Diastasis recti abdominis (DRA) is a prevalent condition in the postpartum period. To date, there is scant knowledge on how DRA influences physical, mental, and emotional health. This study investigates primiparous women`s knowledge about DRA, concerns about abdominal appearance, and perceived abdominal muscle strength, comparing women with and without reported DRA.

**Methods:**

This was a cross-sectional comparison study. Data were collected by a web-based questionnaire, mainly through social media in Norway. To be included in the study women had to be primiparous 6–8 months postpartum. The questionnaire contained questions regarding women`s knowledge about DRA, perceived protrusion, received treatment, concerns with abdominal appearance and muscle strength. Abdominal body image was measured through the shape concern questions from The Eating Disorder Examination questionnaire (EDE-Q 6.0). Demographic and other descriptive variables are presented as means with standard deviations (SD) or as frequencies with percentages. Chi-square test of independence and independent sample t-tests were used to compare differences between women with and without abdominal protrusion for categorical and continuous variables, respectively.

**Results:**

Our sample consisted of 460 women. Knowledge about DRA was reported by 415/440 (94.3%) women. A total of 73.3% reported to have been worried during pregnancy about abdominal appearance postpartum. Mean degree of concern about present abdominal appearance was 5.5/10 (SD 2.4). Almost 80% experienced weaker abdominal muscles than pre-pregnancy. Ninety-six women (20.9%) reported a protrusion along the midline of their abdomen. Significantly more women with protrusion reported weaker abdominal muscles than women without protrusion. The most frequent treatment women with protrusion reported were exercises for the abdominal muscles (92.6%). Mean score on the EDE-Q, shape concern questions, was higher in women with reported protrusion (mean score: 2.37 (SD 1.6) than women without protrusion (mean score: 2.14 (SD 1.4), p = 0.175.

**Conclusion:**

Primiparous women are concerned about abdominal appearance both during pregnancy and after birth. Those reporting abdominal protrusion are less satisfied with their abdominal appearance and they report weaker abdominal muscles than women without protrusion. This study may contribute to improved knowledge about women`s health concerns, and assessment of DRA should be part of routine follow-up of postpartum women.

## Background

Pregnancy and childbirth bring anatomical and morphological changes to the lower back, pelvic girdle, abdomen and pelvic floor [1]. The most obvious change is related to growth of the fetus and stretching of the abdominal muscles, potentially influencing the mother’s posture and balance [1]. A possible link between injuries and weakness of the abdominal muscles and pelvic floor dysfunction (defined as urinary incontinence, anal incontinence, and pelvic organ prolapse), low back and pelvic girdle pain has been suggested, but results are conflicting[2, 3].

Diastasis recti abdominis (DRA) is defined as an impairment with midline separation of the two rectus abdominis muscles along the linea alba, and is diagnosed by measuring the distance between the medial border of the two rectus abdominis muscles, the inter-recti distance (IRD)[4, 5]. Candido, Lo, and Janssen (2005) [6] judge DRA as present if women present with protrusion/bulging during a crunch, and protrusion during physical activity is considered an important sign of a more severe DRA [7]. DRA affects a significant number of women during the antenatal- and postnatal period and postpartum the prevalence rates of DRA vary between 30 − 68% [8, 9]. In a longitudinal study of 300 primiparous women who gave birth at a public hospital in Norway, Sperstad, Tennfjord, Hilde, Ellstrom-Engh, and Bø (2016) [10] found that prevalence rates changed from 60% 6 weeks postpartum to 45.4% and 32.6%, 6 months and 12 months postpartum, respectively. Impaired abdominal strength in women with DRA has been reported [11–13], but a systematic review concluded that the evidence for such associations was weak [2]. Strength training of the abdominal muscles is one proposed method to treat DRA, yet there is currently very low-quality scientific evidence to recommend specific exercise programs in the treatment of DRA postpartum [14].

Recent research has documented a connection between increased media attention and body image concerns among pregnant and postpartum women [15–17]. A systematic review and meta-synthesis of women`s experiences in pregnancy and postpartum body image found that body dissatisfaction dominated the postpartum period and that women may have unrealistic expectations for their postpartum body [18]. Also, a study exploring appearance-related images and messages in pregnancy magazines found that a substantial portion of advertisements promoted products for postpartum weight loss. The research concluded that these magazines may contribute to body dissatisfaction [19]. A recent systematic review found that the focus for investigation on DRA had been on associations with physical challenges such as pelvic floor dysfunction, low back and pelvic girdle pain, and there were few studies on associations with body image, physical appearance and body satisfaction [20].

The aims of the present study were to investigate primiparous women`s knowledge about DRA, whether they have concerns about abdominal appearance and, perceive impaired abdominal muscle strength 6–8 months postpartum. Further to study whether there are differences between women with and without reported abdominal protrusion regarding knowledge about DRA, concerns about abdominal appearance, and perceived abdominal muscle strength 6–8 months postpartum.

## Methods

This was a descriptive cross-sectional comparison university initiated and conducted study. The study was conducted between March 2019 and August 2020. Healthcare clinics in Oslo and Akershus county, Norway and social media (Facebook and Instagram) were used to recruit women. The participants signed up by sending an email to the researcher or by clicking on a registration link. Before inclusion they had to confirm that they fulfilled the inclusion criteria. They subsequently received an email with the informed consent and a link to the electronic questionnaire.

Inclusion criteria were primiparous women 6–8 months postpartum with a single or multiple pregnancy, any mode of delivery, and who were able to understand a Scandinavian language. Exclusion criteria were multiparous women, being < six months and > eight months postpartum, and being < 18 years old. In addition, we excluded responses with no answers, duplicates and no postpartum data.

The participants were invited to respond to an electronic survey (SurveyXact) via their personal phone or computer. Up to three reminders were sent to non-responders.

The questionnaire was a new web-based questionnaire developed for the present study, containing a combination of validated instruments and new questions developed from a focus group of a convenient assembly of parous women. The response options for the new questions were a mix of 11-point Likert scales, close-ended, and semi-close-ended questions. The new questions and response categories were piloted among members of our research group and women in the focus group, and the questions were revised accordingly for clarity.

Response to all 162 questions in the questionnaire required a maximum of 30 min to complete. However, less time was required for those who reported no abdominal, low back-, or pelvic girdle pain, or pelvic floor dysfunction.

### Demographic variables

Participants` demographics included age, height, pre-pregnancy weight, current weight, weight gain in pregnancy, single/twin pregnancy, time since birth, mode of delivery, child`s birth weight and length, physical activity level (frequency and min/week) and self-reported health, smoking habits, workload, breastfeeding, menstrual cycle, education level, and ethnic origin.

The questions about exercise frequency and exercise duration were from The Physical Activity and Pregnancy Questionnaire, which has been found to be an acceptable measure of habitual physical activity and exercise among pregnant women at group level [21]. Questions about self-reported health were from the Norwegian Mother and Child Cohort Study, available at www.fhi.no/morogbarn. We have not been able to find specific questionnaires on physical activity in the postpartum period.

### Knowledge about DRA

- Have you heard about separation of the abdominals/ DRA related to pregnancy or the postpartum period? Response options: “yes” or “no”.

Only women responding yes to the above question, were asked to respond to the following questions;

- Where did you hear about DRA? Response options: “scientific literature”, “the health service system (doctor, midwife, nurse, physiotherapists)”, “friends/ acquaintances”, “social media”, “TV/ media”, “women’s magazines”, or “other”. Multiple response categories were allowed.
- Have you tried one or more treatments to decrease the separation between your abdominals postpartum? Response options: “yes” or “no”.

Only women responding yes to the above question were asked to respond to the following questions;

- What type of treatment have you tried to resolve DRA? Response options: “surgery”, “application of specific creams to the abdomen”, “external support/corset”, tape (e.g. kinesio tape)”, “electrical stimulation”, “exercises for the pelvic floor muscles (PFM)”, “exercises for the abdominals”, or “other”. Multiple response categories were allowed.
- From where/whom have you found/received treatment/exercises for DRA? “internet sites/ social media”, “physiotherapist”, “naprapath/chiropractor/osteopath”, “fitness center/ personal trainer”, or “others (family/friends)”. Multiple response categories were allowed.
- How would you describe your DRA now compared to before treatment? The participants rated their recovery on a scale from − 5 to 5, where − 5 represented “very much worse”, 0 represented “unchanged”, and 5 represented complete recovery.

### Abdominal protrusion, appearance and perceived abdominal muscle strength

All women responded to how they perceived their abdominal appearance using categorical responses and numeric rating on scales from 0 to 10.

- Do you experience a protrusion along the midline of your abdomen? “no, never”, “yes, sometimes”, “yes, all the time”, or “do not know”. This variable was dichotomized to yes and no, where “do not know” was classified as no.
- How would you describe the strength of your abdominal muscles? “stronger than pre-pregnancy”, “the same as pre-pregnancy”, “somewhat weaker than pre-pregnancy”, “much weaker than pre-pregnancy”, or “do not know”.
- Do you feel that the skin on your abdomen is flabby/lax? “no”, “yes, somewhat”, or “yes, to a great extent”.
- Have you developed striae on your abdomen during pregnancy/postpartum? “no”, “yes, a few”, “yes, several”, or “yes, a lot”.

Only women responding yes to flabby/lax skin and/or striae rated bothersomeness on a scale from 0 to 10 where 0 represented “not at all” and 10 “to a great extent”. In addition, all women answered the following questions;

- Have others (e.g. friends or family) been concerned about your abdominal appearance postpartum? “no”, “yes, to some degree”, or “yes, to a great extent”. In the comparison analysis between women with and without reported protrusion this variable was dichotomized into “yes” (to some degree/ great extent) and “no”.
- Do you find there is too much focus from the media, TV, internet, magazines, about having a flat abdomen postpartum? “strongly disagree”, “disagree”, “neither”, “quite agree”, or, “agree to a great extent”. In the comparison analysis between women with and without reported protrusion this variable was dichotomized into “yes” (quite agree/agree in great extent) and “no” (neither/disagree/strongly disagree).
- While you were pregnant, were you worried about how your abdomen would look postpartum? “no”, “yes, to some degree”, “yes, to a great extent”. In the comparison analysis between women with and without reported protrusion this variable was dichotomized into “yes, worried” (some degree/great extent) and “no”.
- To what extent are you concerned about your abdominal appearance today on a scale from 0 to 10 where 0 represents “not at all” and 10 “to a great extent”?
- Overall, how satisfied are you with your abdominal appearance postpartum on a scale from 0 to 10 where 0 represents “very dissatisfied” and 10 “very satisfied”?

### Abdominal body image

- The Eating Disorder Examination questionnaire (EDE-Q 6.0) is a self-reported measure of eating disorder psychopathology focusing on the previous 28 days and consisting of 28-item divided into four subscales. The Norwegian EDE-Q version has shown good test-retest reliability with a Spearman’s correlation coefficients of 0.82–0.91 for the subscales. Norms for the subscale; shape concern for healthy women is 1.8 (SD 1.6) and 4.7 (SD 1.4) in women with an eating disorder [22]. Due to a possible link between abdominal appearance and the shape concern questions, and for the purpose of the present study, only the eight questions from EDE-Q 6.0; shape concern, were included to assess abdominal body image [23].

All statistical analyses were performed using SPSS statistical software package version 24 (SPSS Inc., Chicago IL, USA). Demographic and other descriptive variables are presented as means with standard deviations (SD) or as frequencies with percentages. Shapiro-Wilk test was used to test normality of distribution. Paired sample t-tests were used to compare the participants mean score for continuous variables pre-pregnancy, during pregnancy and postpartum. Chi-square test of independence (with Yates Continuity Correction) and independent sample t-tests were used to compare differences between women with and without protrusion for categorical and continuous variables, respectively. P-value was set at < 0.05.

## Results

**Fig. 1:**
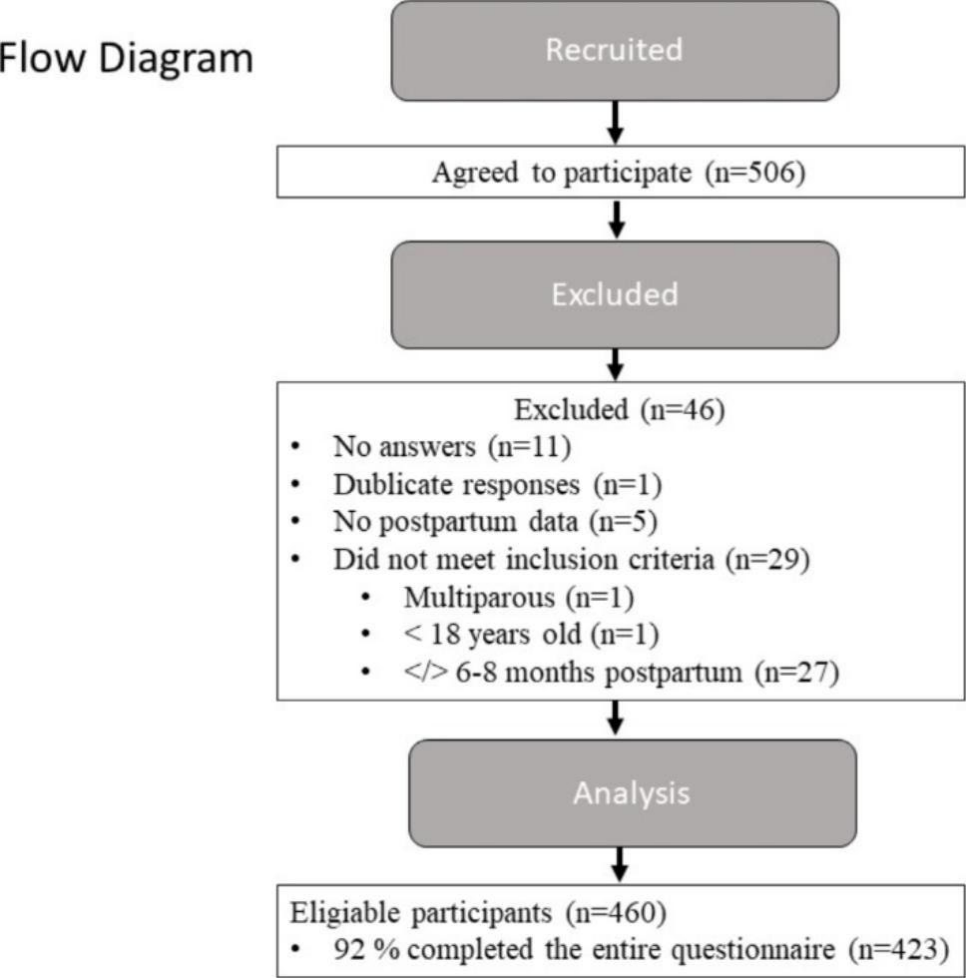
Flow diagram of study particpants

Our sample comprised 460 women, recruited mainly through social media in Norway. Flow diagram of included women with reason for exclusion are presented are Fig. 1.

Table 1 presents the background variables of the participants. Most women were married/cohabitating, had a college/university education and were of Scandinavian origin.

**Table 1:** Characteristics of included primiparous women at 6–8 months postpartum. N = 460

Variable	
Age, years	30.4 (3.6)
Education level	
University High school/college Elementary school Other	400 (87.0) 56 (12.2) 3 (0.7) 1 (0.2)
BMI, kg/m^2 a^	24.5 (4.3)
Weight gain in pregnancy, kg^b^	16.2 (9.8)
Weight pre-pregnancy, kg^c^	67.7 (12.3)
Single pregnancy Twin pregnancy	455 (98.9) 5 (1.1)
Time since birth	
6 months 7 months 8 months	203 (44.1) 150 (32.6) 107 (23.3)
Mode of delivery	
Vaginal Cesarean	378 (82.2) 82 (17.8)
Week of delivery	
Between week 26 and 30 Between week 31 and 36 Week 37 or later	3 (0.6) 38 (8.3) 419 (91.1)
Child`s birth weight, gram	
˃ 4500 4000–4500 3000–3999 2500–2999 1500–2499 1000–1499	10 (2.2) 61 (13.3) 326 (70.9) 49 (10.7) 13 (2.8) 1 (0.2)
Child`s birth length, cm^d^	50.2 (2.9)
Current use of contraceptives	
Yes No	182 (39.6) 278 (60.4)
Current breastfeeding	
˃ 3 times or more/day 1–2 times/day 4–6 times/week 1–3 times/week Rarely/never	352 (76.5) 21 (4.6) 1 (0.2) 3 (0.7) 83 (18.0)
Back to work postpartum	
Yes No	54 (11.7) 406 (88.3)
Heavy lifting at work^e^	
Perform heavy lifting Rarely/never perform heavy lifting	18 (33.3) 36 (66.7)
Physical activity, n/week^f^	
Never < 1/week 1/week 2/week 3–4/week ≥ 5/week	15 (3.4) 46 (10.3) 67 (15.0) 110 (24.6) 151 (33.8) 58 (13.0)
Physical activity, min/week^f^	147 (SD 132.3)
< 149 min/week ≥ 150 min/week	259 (57.9) 188 (42.1)
Self-reported health^g^	
Very good Good Neither good nor bad Bad Very bad	109 (23.7) 248 (54.0) 80 (17.4) 21 (4.6) 1 (0.2)
Smoking	
Yes, every day Yes, rarely No	2 (0.4) 3 (0.7) 455 (98.9)
Menstruating	
Yes No Unknown	195 (42.4) 211 (45.9) 54 (11.5)

The table shows means with standard deviation (SD) or numbers with percentages (%)

^a^Total n = 432; 28 women did not want to answer the question about current weight in the questionnaire

^b^Total n = 451; 9 women did not want to answer the question about weight gain in the questionnaire

^c^Total n = 439; 21 women did not want to answer the question about weight gain in the questionnaire

^d^Total n = 455; 5 women did not enter child length in the questionnaire due to breech birth

^e^Total n = 54 women responded yes to be back to work postpartum

^f^Total n = 447; 13 women did not answer this question (valid percent reported)

^g^Total n = 459; 1 woman did not answer this question (valid percent reported)

### Knowledge of DRA

Knowledge about DRA was reported by 415/440 (94.3%) women. Friends and acquaintances, social media and health personnel were the most common sources of this information. Approximately 20% of the women who knew about DRA had tried one or more treatment options to reduce it postpartum. The most frequently reported treatments were PFM exercises (84.1%) and abdominal muscle exercises (82.9%). These were mostly assessed through social media 44/82 (53.7%). No change in abdominal recovery after treatment was reported in 14/81 (17.3%) women and 23/81 (28.3%) reported complete recovery.

### Abdominal appearance and perceived abdominal strength

Table 2 shows concerns and satisfaction with abdominal appearance and perceived abdominal strength for the total sample. More than 2/3 of the participants agreed or strongly agreed that there is an excessive social media focus on regaining a “flat abdomen” postpartum. Almost 70% reported laxity of the abdominal skin and 33% the development of striae on the abdomen during pregnancy or in the postpartum period. A total of 73.3% of the women were worried during pregnancy about abdominal appearance after childbirth. Mean degree of overall satisfaction with abdominal appearance was 5.8/10 (SD 2.6). When splitting the data into women with a singleton- and twin pregnancy the mean degree of overall satisfaction with abdominal appearance was 5.8 (SD 2.6) and 3.8 (SD 3.6), respectively. Splitting data on the women`s delivery week the mean overall degree of satisfaction with abdominal appearance was 6.7 (SD 1.5) for week 26–30, 5.9 (SD 2.6) for week 31–36 and 5.8 (SD 2.6) for week 37+. Splitting data on the child`s birth weight the overall degree of satisfaction with abdominal appearance was 4.6 (SD 3.4) (˃ 4500 g), 5.0 (SD 2.7) (4000-4500 g), 5.8 (SD 2.5) (3000-3999 g), 6.1 (SD 2.5) (2500-2999 g), 6.9 (SD 3.0) (1500-2499 g), and 8.0 (1000-1499 g).

**Table 2:** Concerns and satisfaction with abdominal appearance and perceived abdominal strength in primiparous women 6–8 months postpartum

	Total sample	Women reporting protrusion	Women without protrusion	Difference between groups, p-value
Worries during pregnancy about abdominal appearance postpartum Yes, to some/great extent No	321/438 (73.3%) 117/438 (26.7%)	76/321 (23.7%) 20/117 (17.1%)	245/321 (76.3%) 97/117 (82.9%)	0.153
Present degree of abdominal appearance concern	5.5 (SD 2.4)^a^	6.16 (SD 2.3)^b^	5.35 (SD 2.4)^c^	0.003 (MD -0.8, 95% CI -1.3,-0.3)
Striae Yes, some/many No, none Bothersomeness of striae	145/438 (33.1%) 293/438 (66.9%) 3.7 (SD 3.2)^d^	27/145 (18.6%) 69/293 (23.5%) 4.11 (SD 3.66)^e^	118/145 (81.4%) 224/293 (76.5%) 3.56 (SD 3.11)^f^	0.270 0.423 (MD -0.6, 95% CI -1.9, 0.8)
Lax skin Yes, in some/great extent No Bothersomeness of lax skin	298/438 (68.1%) 140/438 (31.9%) 4.1 (SD 2.8)^g^	69/298 (23.2%) 27/140 (19.3%) 4.64 (SD 2.95)^h^	229/298 (76.8%) 113/140 (80.7%) 3.97 (SD 2.74)^i^	0.388 0.082 (MD -0.67, 95% CI -1.42, 0.09)
Overall degree of satisfaction with abdominal appearance	5.8 (SD 2.6)^a^	5.29 (SD 2.8)^b^	5.90 (SD 2.54)^c^	0.043 (MD 0.61, 95% CI 0.02–1.19)
Too much focus in social media about flat abdomen postpartum Yes, quite/strongly agree No, quite/strongly disagree or neither	310/438 (70.8%) 128/438 (29.2%)	73/310 (23.5%) 23/128 (18%)	237/310 (76.5%) 105/128 (82%)	0.253
Concerns from family/friends (abdominal appearance) Yes, to some/great extent No	152/438 (34.7%) 286/438 (65.3%)	33/152 (21.7%) 63/286 (22%)	119/152 (78.3%) 223/286 (78%)	1.000
Weaker abdominal muscles than pre-pregnancy. Yes, slightly/very much No, similar/stronger	349/438 (79.6%) 89/438 (20.4%)	90/349 (25.8%) 6/89 (6.7%)	259/349 (74.2%) 83/89 (93.3%)	< 0.001

Values are presented for the total sample and women with and without reported protrusion as means with standard deviations (SD) or as frequencies with percentages (valid percent reported). There are dissimilar numbers of women responding to each question in the table

^a^Total n = 438; 22 women did not answer the questions about abdominal appearance in the questionnaire

^b^Total n = 96 women reported protrusion in the questionnaire

^c^Total n = 342 women did not reported protrusion in the questionnaire

^d^Total n = 145; only responded by women who reported striae in the questionnaire

^e^Total n = 27; women with stria who reported striae in the questionnaire

^f^Total n = 118; women with striae, who reported no protrusion in the questionnaire

^g^Total n = 298; only responded by women who reported lax skin in the questionnaire

^h^Total n = 69; women with lax skin who reported protrusion in the questionnaire

^i^Total n = 229; women with lax skin who reported no protrusion in the questionnaire

Almost 80% reported weaker abdominal muscles after childbirth compared with pre-pregnancy levels.

### Abdominal body image

Four hundred and thirty-three women responded the EDE-Q, subscale shape concern questions. Mean subscale score for included women was 2.19/6.0 (SD 1.48). A mean subscale score above 4.7 was reported in 32/433 (7.4%) women.

### Women reporting abdominal protrusion

Ninety-six women (20.9%) reported a protrusion along the midline of their abdomen. There was no statistically significant difference in background variables between women with and without protrusion. In women who reported protrusion the prevalence of Caesarean section was 31.1% compared to 20.1% of women with vaginal delivery (p = 0.053). One or more treatment methods were tried in 27/92 (29%) of women with reported protrusion. The most frequent reported interventions were exercises for the abdominal muscles (92.6%) and the PFM (81.5%). The exercises were mostly sourced through social media 14/27 (51.9%). 22% of the women with reported protrusion who had tried any form of treatment for DRA (n = 27) reported no improvement and 11% reported complete recovery.

Table 2 shows concerns and satisfaction with abdominal appearance and perceived abdominal strength for women with and without reported protrusion. Women reporting abdominal protrusion were significantly more preoccupied with the appearance of their abdomen and less satisfied with their abdominal appearance. Significantly more women with protrusion reported weaker abdominal muscles compared to women without protrusion.

There was no significant difference in mean score on the EDE-Q shape concern questions in women with reported protrusion (mean score: 2.37 (SD 1.6)) compared to women without protrusion (mean score: 2.14 (SD 1.4)), p = 0.175.

## Discussion

The results of the present study showed that primiparous women are concerned about abdominal appearance both during pregnancy and in the postpartum period and most seek advice of treatment through social media. Women reporting protrusion of the abdominal wall are less satisfied with their abdominal appearance and they report weaker abdominal muscles than women without protrusion. In addition, those reporting abdominal protrusion report weaker abdominal muscles than women without protrusion.

### Knowledge of DRA

Most of our study participants knew about DRA and this information mostly came from social media. Eriksson-Crommert, Petrov Fieril, and Gustavsson (2020) [24] found that women with DRA reported a lack of understanding of their condition and that the health care system showed little interest and insufficient knowledge of the condition. This underpins the search for information through social media, and social media may therefore have impacted our participants’ expectation regarding their abdominal shape postpartum. Although they report to be concerned about the protrusion of their abdominal wall, few women had searched for treatment within the health care system. Zhu et al. (2019) [17] confirmed a shift in pregnancy-related information seeking from caregivers to social media. Gustavsson and Eriksson-Crommert (2020) [25] reported no consensus among health care professionals on how to best approach DRA, and that health personnel also used social media and other webpages to seek knowledge of the condition and treatment options.

We found that more than 2/3 of our participants agreed or strongly agreed that there is too much media focus on regaining a “flat abdomen”. Studies have shown that media influence on body image is common in women and adolescent girls[26, 27]. Coyne et al. (2018) [15] reported lower body image in pregnant women after only five minutes of exposure to magazines containing glamorized media portrayals of pregnant/postpartum women, compared to women reading a control magazine. Although regular physical activity and regaining pre pregnancy weight may have several advantages for health in the postpartum period [28], the postpartum period can be a vulnerable period for the women’s self-esteem and body image. A systematic review and meta-synthesis of women`s experiences in pregnancy and postpartum body image found that body dissatisfaction dominated the postpartum period and that women may have unrealistic expectations for their postpartum body [18]. It is therefore important to share evidence-based information on the natural remission of DRA during the first year postpartum on social media [10].

### Abdominal appearance and perceived abdominal strength

Although several studies have investigated how postpartum women feel about their body[18, 29], as far as we have ascertained this is the first study to ask specifically about satisfaction and concerns with abdominal appearance, striae and loose skin. We found that mothers are concerned and dissatisfied with abdominal appearance. This adds to existing data from a systematic review and meta-synthesis showing that body dissatisfaction dominated the postpartum period and that women may have unrealistic expectations for their postpartum body [18]. Rallis, Skouteris, Wertheim, and Paxton (2007) [30] found that 6 months postpartum was when women reported most concern about their body. This confers with the time period of our study. Whether further recovery may occur after this time period or whether women later accept the postpartum body needs further investigation. In addition, we found that women who gave birth at term or had a normal weight child were less satisfied with their abdominal appearance compared to those giving birth preterm or having an underweight child. Also, women who had a twin delivery where less satisfied compared to women with a singleton pregnancy.

Almost 80% of our sample reported weaker abdominal muscles. This is based on women`s perceptions only, and not clinical assessment. There are few clinical studies of abdominal function after birth and there is a great diversity in how the studies assess abdominal muscle strength. Gilleard and Brown (1996) [31] assessed the functional capability of the abdominal muscle group to stabilize the pelvis against resistance. Six primiparous women were assessed < 8 weeks postpartum and the authors found decreased abdominal muscle function in the early postpartum period. Hills et al. (2018) [12] reported an association between ability to perform a sit-up and trunk rotation strength and DRA in primiparous women 1 year postpartum, while the result of Gluppe et el. (2021) [3] did not confer with these results. Benjamin et al. (2018) [2] concluded in 2018 with week evidence, and there is still a need for further studies to understand the influence of DRA on muscle strength.

### Abdominal body image

The mean subscale score for shape concern the total sample in our study was higher than the mean subscale score for healthy Norwegian women (n = 1845); 1.8 (SD 1.6) [22]. Although we only used the shape concern questions from the EDE-Q 6.0, our results are in line with the results of the general postpartum population. Our result was not statistically significant, but we found a higher mean score for the shape concern questions in women with reported abdominal protrusion than in women without. Eating disorder symptoms is prevalent in the postpartum period [32–34]. The present study`s reported subscale score for women with a possible DRA is high and might therefor indicate a possible clinical eating disorder. However, we only used the shape concern questions of the EDE-Q 6.0 and can therefore not report on eating disorder in our population. We suggest this is an important aspect to include in future studies and in the follow up of women with DRA postpartum.

### Women reporting abdominal protrusion

In the present study there was no clinical assessment with observation of protrusion. Self-report of DRA and protrusion may be considered less valid than assessment by health personnel. However, the prevalence of 26.5% in women reporting abdominal protrusion in the present study is in line with previous research, although a bit lower than was found in a clinical study from the same country [10]. Sperstad et al. (2016) [10] included only primiparous women, pointing towards the likelihood of underestimation of the prevalence of DRA in our study. Due to natural remission of DRA in about 30% of women during the first year postpartum [10], we chose to include women 6–8 months postpartum. We also wanted to be able to compare reported prevalence of protrusion with other studies that evaluated women at this time-point.

We found that women reporting protrusion reported significantly weaker abdominal muscle strength postpartum compared to pre-pregnancy. Several clinical studies comparing women with and without DRA confer with these results[11, 12, 35]. However, in a recently published study by Gluppe et al. (2021) [3] no significant difference in abdominal muscle strength was found in the adjusted analysis of women with and without DRA diagnosed with ultrasound. To date there is no consensus on how to best capture abdominal muscle strength, and the published studies have used different methods to assess it. Direct comparison between studies is therefore not possible.

We found no difference in bothersomeness about striae or lax skin between women with or without reported protrusion. However, women with reported protrusion showed a tendency of more lax skin than women without reported protrusion. Lax skin may be a marker of weak connective tissue and subsequent risk factor for DRA [36]. Further basic studies are needed to investigate mechanisms for striae, lax skin and DRA.

The findings of less satisfaction with abdominal appearance in women reporting protrusion confer with a qualitative study finding that women with increased IRD might experience body dissatisfaction [24]. Also, Keshwani et al. (2018) [37] reported a significant correlation between IRD and body image. In addition, the same research group reported that a physiotherapy intervention had a positive effect on body image in women with DRA [38]. A systematic review on self-reported symptoms in women with DRA did not find any other studies reporting on body image in women with DRA [20]. Interestingly, most women in our study had followed treatment programs found through social media. However, although advocated as highly effective, these programs have not been tested in randomized controlled trials, and the effect is therefore unknown. Women with reported protrusion in our study had mostly used programs containing exercises for the abdominals or the PFM, or a combination of both in the treatment of DRA. This is in accordance with a study from the United States showing that women`s health physiotherapists reported exercises for transversus abdominis (89%) and PFM (87%) as treatment for women with DRA [39]. A recent systematic review of efficacy of different abdominal and PFM training concluded with very low-level evidence that transversus abdominis training is more effective than minimal intervention, and low to very low-level evidence that PFM training is not more effective than minimal intervention for treating DRA[14, 40]. Based on our findings, we agree with Fuentes Aparicio et al. (2020) [20], who suggested inclusion of perception of body image in future studies of treatment of women with DRA.

As response rate and generalizability is not possible in web-based surveys, we may compare our participants with the official statistics of the Norwegian Institute of Public Health`s Medical Birth Register. In 2019 54 407 births were registered. 43% of these were primiparous, 98.5% were single deliveries and 15.9% gave birth by Cesarean section. Mean age in primiparous women was 29.7 years (SD 4.8), women’s mean BMI prior to pregnancy was 24.6 (SD 4.9) and the baby`s mean birth weight was 3500 g (SD 592). Our sample is comparable to the official characteristics of these background variables in Norwegian primiparous women, which may be considered a strength of the study. A limitation is that no power calculation was performed for the present study, however we consider the study`s sample size a major strength and that 92% responded to the entire questionnaire. Further, the use of DRA with a protrusion as an inclusion criteria for being classified with DRA in comparison with no DRA, may indicate a more robust diagnoses compared with a simple question on whether the women experience DRA. Inclusion of women with twin pregnancy, delivery between the 26th and 30th gestational week, and child`s birth weight (1000-1499 g) might be considered a limitation. However, the numbers of women in these groups are few and we therefore decided to include them and report the result of these subgroups separately. The results may serve as background for future studies.

Although background variables of the participants are comparable with the total population, we cannot ensure generalizability as selection bias may have occurred due to recruitment mainly through social media as more women with more concerns about this topic might have been recruited. Our participants had a high educational level. Norway has a high uptake of mobile phones, and access to internet and social media is also commonly used by new mothers in other countries [17], but this may not be the case in all societies. Another limitation is lack of clinical assessment both of DRA and muscle strength. The use of a questionnaire allowed us to include a large sample but asking about protrusion may have underestimated the number of women with DRA. Thus, this can be both a strength and a limitation. In addition, the term protrusion may have been interpreted differently among participating women, and the prevalence of women with DRA in our study may be both over or underestimated.

## Conclusion

Our study found that primiparous women are concerned about abdominal appearance and that women reporting DRA with protrusion experience reduced abdominal strength in comparison with pre-pregnancy level. In the postpartum period women learn about exercises for the abdominals from social media where there is no quality assurance of the information. There is a need for future follow-up assessments of women in the postpartum period concerning DRA, abdominal strength, and abdominal body image. Further high quality RCTs on the effect of different exercise programs in prevention and treatment of DRA are warranted to be able to guide women returning to exercise after childbirth.

## Data Availability

The datasets used and/or analyzed during the current study are available from the corresponding author on reasonable request.

## Abbreviations

DRA: Diastasis recti abdominis.
EDE-Q: The Eating Disorder Examination questionnaire.
IRD: inter-recti distance.
PFM: Pelvic floor muscles.
SD: Standard deviations.
